# Long non-coding RNA: its evolutionary relics and biological implications in mammals: a review

**DOI:** 10.1186/s40781-018-0183-7

**Published:** 2018-10-25

**Authors:** Jasdeep Kaur Dhanoa, Ram Saran Sethi, Ramneek Verma, Jaspreet Singh Arora, Chandra Sekhar Mukhopadhyay

**Affiliations:** School of Animal Biotechnology, Guru Angad Dev Veterinary and Animal Sciences University, Ludhiana, Punjab India

**Keywords:** Long non-coding RNA, Biogenesis, Disease biomarker, Evolution, Mammals

## Abstract

The central dogma of gene expression propounds that DNA is transcribed to mRNA and finally gets translated into protein. Only 2–3% of the genomic DNA is transcribed to protein-coding mRNA. Interestingly, only a further minuscule part of genomic DNA encodes for long non-coding RNAs (lncRNAs) which are characteristically more than 200 nucleotides long and can be transcribed from both protein-coding (e.g. *H19* and *TUG1*) as well as non-coding DNA by RNA polymerase II. The lncRNAs do not have open reading frames (with some exceptions), 3`-untranslated regions (3’-UTRs) and necessarily these RNAs lack any translation-termination regions, however, these can be spliced, capped and polyadenylated as mRNA molecules. The flexibility of lncRNAs confers them specific 3D-conformations that eventually enable the lncRNAs to interact with proteins, DNA or other RNA molecules via base pairing or by forming networks. The lncRNAs play a major role in gene regulation, cell differentiation, cancer cell invasion and metastasis and chromatin remodeling. Deregulation of lncRNA is also responsible for numerous diseases in mammals. Various studies have revealed their significance as biomarkers for prognosis and diagnosis of cancer. The aim of this review is to overview the salient features, evolution, biogenesis and biological importance of these molecules in the mammalian system.

## Background

The organization of eukaryotic genome is very complex. Almost 98% of the human genome does not encode proteins [109]. This non-coding DNA was assumed to be a “barren land” with no apparent functionality in protein synthesis and thus erstwhile it was termed as “junk DNA” [50, 73, 75]. However, the non-coding, intergenic DNA was later found to be a treasure of information that can be deciphered in the form of nucleotide elements (repetitive, transposable, interspersed elements etc) and different non-coding RNAs (rRNAs, tRNAs, regulatory RNAs etc). The RNA molecules lacking protein-codingcapacity are known as non-coding RNAs (ncRNAs). How much non-coding sequences are functional is still a matter of debate. The reports published by Encyclopedia of DNA elements (ENCODE) revealed that approximately 80.4% of the genome is involved in some sort of biochemical activity including chromatin structure, histone modification and RNA transcription etc. [71]. The non-coding transcripts less than 200 bases are called small non-coding RNA and comprise of tRNA, rRNA, miRNA, snoRNA, piwi-interacting RNA (pi-RNA) etc. [47]. The proportion of different ncRNAs to the total amount of RNA in a healthy eukaryotic cell, other than rRNA (80–90%) and tRNA (10–15%), ranges between 0.002 to 0.2% [15]. On the contrary, RNA molecules that are of more than 200 bases in length are known as long non-coding RNA (lncRNA) [82].

The lncRNAs and other non-coding RNAs including miRNAs (21–24 bases) and piRNAs (26–31 bases) are involved in epigenetic modification of DNA, and regulation of transcriptional and post-transcriptional gene expression [25, 69]. In the course of time, different non-coding RNAs (antisense RNAs, snoRNAs, miRNAs, piRNAsetc) have been discovered in animals and plants. Of late, considerable research emphasis has been given towards lncRNAs and their diverse role in various diseases in animals, especially human and mice. H19, an imprinted long non-coding RNA gene that encodes an untranslated RNA, is transcribed only from maternally inherited alleles. This feature is responsible for its role as a negative regulation of body weight and cell proliferation. The maternal disruption of this gene in mice showed somatic overgrowth of heterozygotes whereas no effect was noticed during disrupted paternal inheritance [57]. The study showed that transgenic mice lacking functional H19 exhibit normal development, however, other experiments in mice revealed that overexpression of H19 affects their prenatal viability [43, 49]. The lncRNAs exercise a very wide variety of functions in animals, which have been discussed later in this review. Certain lncRNAs are reported to be associated with counteracting toxic conditions in the human body. The lncRNAs associated with toxicological responses to various xenobiotics (Benzene, Phenobarbital, Cadmium etc.), in human, has also been reported [14].

The lncRNAs exhibit their biological functions by acting as *cis-* or *trans-* regulators in biological processes [63, 79, 95]. The lncRNAs that control chromatin structure interact with nucleosome remodeling factors as well as chromatin modifying enzymes [33]. Such long non-coding RNAs usually have limited coding potential due to the absence of open reading frames, 3`-UTR and termination region. In this review, we are going to outline the literature findings of the basic features, functions and differential role of lncRNAs in the biological system.

## Features of lncRNA

### Length

As discussed above, the non-coding transcripts that do not encode proteins and are more than 200 nucleotides in length are known as long non-coding RNAs (lncRNAs). The length of a lncRNA can be more than 2 Kb while their coding potential is less than 100 amino acids [5]. Kaur and colleagues showed that in the human genome 20% of the transcriptional progress would be associated with protein-coding genes. This information illustrates that lncRNAs are four times longer than the coding RNA sequence [5].

### Location in genome

The lncRNAs are harbored mostly in poorly conserved regions in the genome including the intronic regions of genes [51]. Besides, some lncRNAs are reported to be transcribed from one of the strands of a DNA sequence [61] within the protein-coding locus. The genomic locations of the lncRNAs bear direct association with their evolutionary conservedness [52, 53]. Research findings and scientific discussions suggest that plethora of lncRNAs are evolutionarily conserved [54] howbeit to lesser extent as compared to that of the protein-coding genes [55]. Interestingly, the promoter-regions of the lncRNAs are more conserved as compared to the sequence of the lncRNAs [56]. The presence of open reading frames in some lncRNAs makes these molecules difficult to distinguish from protein-coding RNAs [17]. The lncRNA gene ‘X Inactive Specific Transcript’ (or Xist), responsible for X-chromosome inactivation, is an example of lncRNA located within a less conserved region in the genome [81].

### Action

Different families of lncRNAs exercise varying modes of action for gene expression regulation and protein synthesis. These non-coding RNAs (ncRNAs) can act as scaffolds in sub-nuclear domains or can possess secondary structures to interact with DNA, RNA, and protein (http://www.exiqon.com/lncRNA). Long non-coding RNAs havecell-specific expression.It has been reported that transcription of individual lncRNAs occurs at a specifictime; hence they can serve as molecularsignals to respond to diverse stimuli [103].

### *Cis-* and *trans*-regulating action

The specific category of RNAs that exhibit sequence-complementarity to other RNA transcripts is known as natural antisense transcripts (NATs). The *trans*-NATs and their respective targets are physically located in different loci on the genome, like miRNAs. While the *cis*-NATs and their targets are located on the same locus, but opposite strands of the DNA. These cis-NATs were firstly identified in viruses, then prokaryotes and finally in eukaryotes. In eukaryotes (except nematodes), approximately 5–29% of the transcriptional units are involved in the overlap [51]. The *cis-*NATs are transcribed by RNApolymerase II which shows its involvement in mRNA processing. The interaction of sense and antisense transcripts suggests the role of NATs in gene expression regulation. Besides that, it has also been reported that in case of RNA hybrid formation and transcription of gene locus in both orientations can also induce gene silencing or can trigger an immune response [108].

### Comparison with miRNA

miRNAs and lncRNAs, both are non-coding in nature. miRNAs are ~ 22 nucleotides long as compared to 8–10 times longer lncRNAs. The exact functions of lncRNAs are not clear yet but it has been reported that both miRNA and lncRNAs act as regulators for controlling biological processes at post-transcriptional repression of protein-coding genes [101, 102, 105]. Besides,lncRNAs can also act as miRNA sponges and can reduce their regulatory effect on mRNA [78]. Experimental detection of the human genome has identified approximately 2000 different miRNAs and around 50,000 lncRNAs [15, 21, 34].

## Classification of lncRNAs

**Fig. 1:**
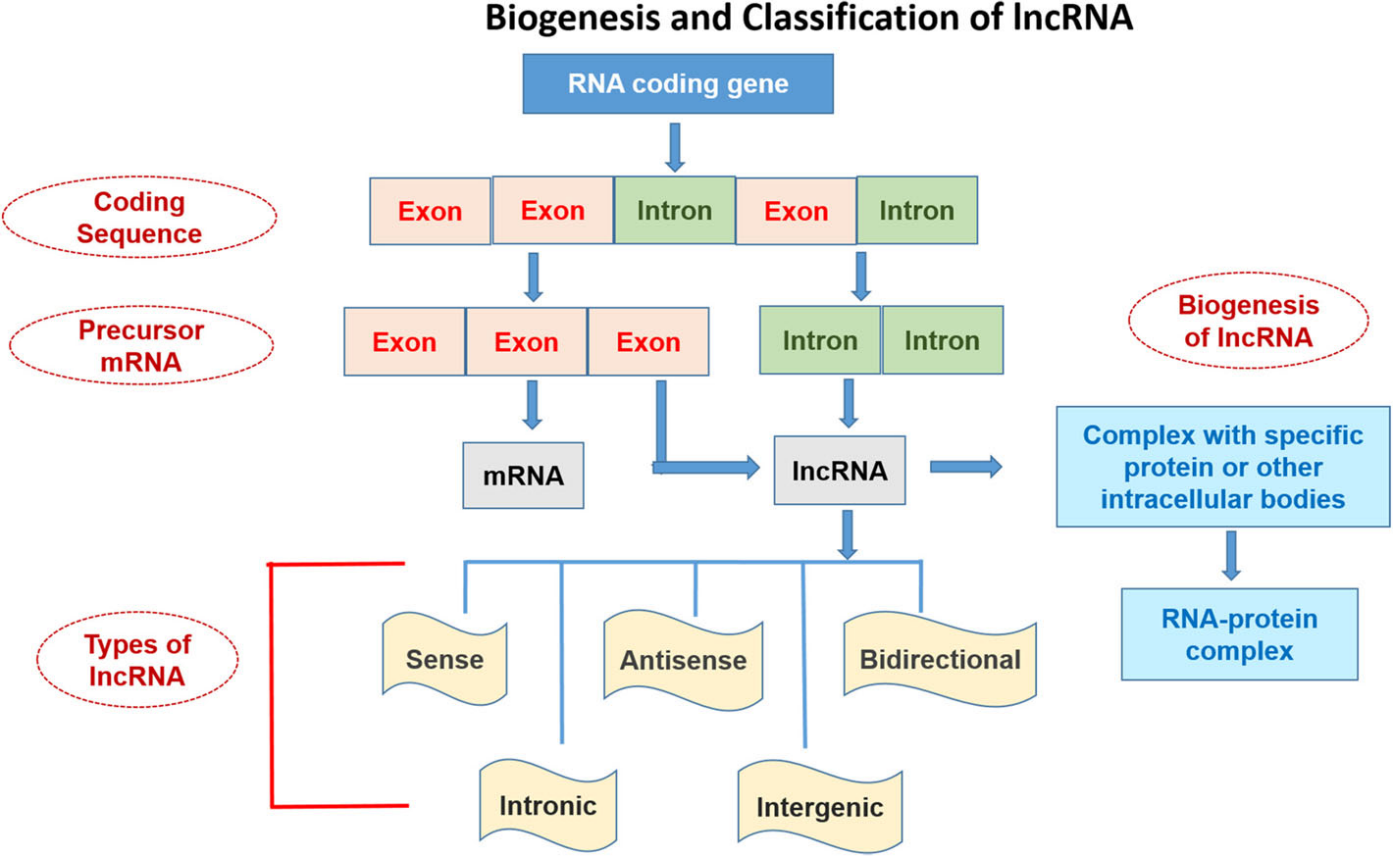
Biogenesis and classification (on the basis of localization) of lncRNAs in humans and other animals

The nomenclature and symbols of 319 human-lncRNAshave been approved by the HUGO-Gene Nomenclature Committee (HGNC) (https://www.genenames.org/cgi-bin/genefamilies/set/788). The lncRNAs are classified on the basis of structure, function, localization, metabolism, and interaction with protein-coding genes or other DNA elements [4]. Secondary and tertiary structures of lncRNA are greatly conserved as compared to its primary structure. The structure-function relationship study of these high molecular weight molecules is challenging because they are difficult to crystallize [58, 59]. Broadly, the lncRNAs can be divided into 5 categories (Fig. 1):

- sense lncRNAs
- antisense lncRNAs
- bidirectional lncRNAs
- intronic lncRNAs and
- intergenic lncRNAs

The aforementioned diversified function classifications can be clustered according to the 3 different modes of regulation. Firstly, as a *competitor*, it can bind to DNA binding proteins and can inhibit their attachment to the target (viz. transcription factors). For example, lncRNA can affect DNA methylation by inhibiting binding of DNMT1 to target DNA that ultimately affects transcriptional activation of the target gene [38]. Secondly, as a *recruiter*, lncRNA can reinforce DNA methylation by recruiting epigenetic modifiers to some target sites [113]. Lastly, lncRNA can act as a *precursor of miRNA* through digestion with some RNases such as Dicer [45].

## Biogenesis of the lncRNAs

The lncRNA can be transcribed from intergenic, exonic or the distal protein-coding regions of the genome by the enzyme RNA-polymerase II (Fig. 1). Then the pre-mature lncRNA gets 3`-polyadenylated and capped on the 5′-end with methyl-guanosine [60]. Often it undergoes alternative splicingwhich is essential to generate protein diversity [26]. The mechanism of alternative splicing can be classified in three ways. Firstly, lncRNAs interact with specific splicing factors and then form RNA-RNA duplexes with pre-mRNA molecules and finally, they affect the chromatin remodeling, thus complete the splicing of target genes [87]. For example, LINC-HELLP, a 205 kb-lncRNA, which is suggested to be involved in pregnancy-associated disease HELLP and splicing regulation. The purification and mass spectrometry experiments revealed that splicing components (including the splicing-related factors Y-Box Binding Protein 1 (YBX1), and Poly(RC) Binding Proteins 1 and 2) and the ribosomal machinery recognizesthis lncRNA. The molecular mechanisms of splicing regulation by this lncRNAare not clear yet, but it was demonstrated that due to mutations in HELLP patients, some portion (5′-end up to the middle) of the LINC-HELLP transcript loses its ability to interact with its protein partners. On the other hand, binding increases with mutations at the far 3′-end [87]. There are some exceptions of functional lncRNAs which are not polyadenylated viz. antisense,‘as-*Oct4-pg5*’ and brain associated‘*BC200*’ [11, 35]. In general, lncRNA-encoding genes consist of own promoters and have their transcription factors (TFs) and unique DNA motifs [14].

Epigenetic modification plays role in lncRNA biogenesis. Histone-methylation plays a major role in transcriptional regulation. Histone H3 lysine 4 (H3K4) methylation is the symbol of transcription activation whereas H3K27 tri-methylation indicates gene silencing. Various lncRNAs including *HOTTIP, XIST, FIRRE* etc. are involved in transcriptional gene activation and organization of 3D nuclear architecture [14]. On the other hand, the decoys of lncRNAs such as Alu transcripts or lncRNA-DNA triplex can inhibit the transcriptional regulation through binding to RNA polII [64]. Binding of different transcription factors (TFs) to lncRNA forms a nascent transcript which ultimately regulates mRNA processing through alternative splicing. This binding of lncRNAs to mRNA can increase or inhibit translation or can promote mRNA decay [6]. Experimental data from small RNA deep sequencing (sRNA-Seq) has suggested that lncRNA can encode small functional RNA too [41]. Mature lncRNAs can be present in the nucleus and/or cytoplasm [80]. Despite the fact that the cytoplasmic lncRNAs are not translated, but small peptides have been identified that were generated from lncRNAs through their association with ribosomes [30]. Some findings indicate that transcriptionally active pseudogenes can also produce these molecules or they can also be transcribed from the promoter or intergenic regions [25].

## Divergent functions of lncRNAs

Most of the lncRNAs, although non-coding in nature, have a diverse role in disease and biological developmental processes. The exact function of lncRNA and its mode of action warrants in-depth study. However, in general, lncRNAs are found to play important role in gene expression regulation of various diseases including cancer. The lncRNA can implement its function in four different ways [3]:

### Signals

The production and presence of signal factors of lncRNAs are an indicator of their transcriptional activity (e.g. KCNQ1ot1 and Xist) [19]. Some lncRNA transcripts such as CCND1 activate or deactivate the natural functions of target protein targets (that are allosterically modified) via intrinsic catalytic activities [106].

### Decoys

Molecular decoys (viz. Gas5, PANDA etc) are polynucleotides that negatively regulate an effector by preventing access of regulatory proteins to DNA. Gas5 is a hairpin-structured lncRNA (resembles glucocorticoid receptors of DNA) that act as a decoy during growth factor starvation. It releases the receptors of DNA during starvation condition and prevents the transcription of metabolic genes [85].

### Guides

The lncRNAs are required for proper localization of specific proteins including ribonucleoprotein complexes. Homeobox antisense intergenic RNA (HOTAIR) is an example of guide lncRNA to localize polycomb repressor complex2 (PRC2) in developmental and cancer-related gene expression. It is associated with tumor invasiveness and metastasis in gastrointestinal, liver, breast and pancreatic cancers [27].

### Scaffolds

The lncRNAs can serve as adaptors to bind more than 2 protein partners, thus are involved in structural roles. The telomerase RNA TERC (TERRA), an example of RNA scaffold, is responsible for telomerase function [99].

Apart from the aforementioned functions, lncRNAs have been reported to be functional in some substructures of mouse brain [66], and have some role associated with transcriptional factors involved in conferring pluripotency to cells [16]. Long intergenic non-coding RNAs (lincRNAs) are lncRNAspresent in the intergenic regions and have an important role in the maintenance of a pluripotent state of cells. Study on mice embryonic stem cells revealed that knockdown of lincRNA gene effects on gene regulation [85].

## Evolutionary perspective

RNA has evolved earlier than DNA as a genetic material. The former has served as a temporary storage of genetic information [20], while the latter confers structural stability to RNA as a double-strandedmolecule and is able to store genetic information [20, 74]. However, RNA retained the diverse array of functionality in deciphering the genetic scripts and encoding proteins.

Iyer and colleagues estimated that more than 10,000 lncRNA coding genes are present in the human genome and about 60,000 lncRNAs are transcribed in all types of cells [40]. Whole genome alignment of human and mouse lncRNAs showed that the exonic region of lncRNAs evolved at a slower rate as compared to those from the intergenic region and introns of protein coding sequence. This indicates that some lncRNAs may be non-functional or their function can overcome precise sequence constraints [36]. The degree of nucleotide conservation of lncRNAs can be examined on inter-specific or intra-specific levels[31].

Bioinformatics support is now available to study evolutionary perspectives of lncRNAs. The software named “*slncky”* has recently been designed for the evolutionary analysis of lncRNA in mammals*.* It uses RNA sequencing data, removes the overlapping transcripts of annotated and unannotated protein-coding genes present in same species and aligns identified transcripts. The remaining set of fragments are characteristically conservedandlong non-coding transcripts, hence these areidentified as lncRNAs [10].

## Biological role of lncRNAs: Association with diseases

**Fig. 2:**
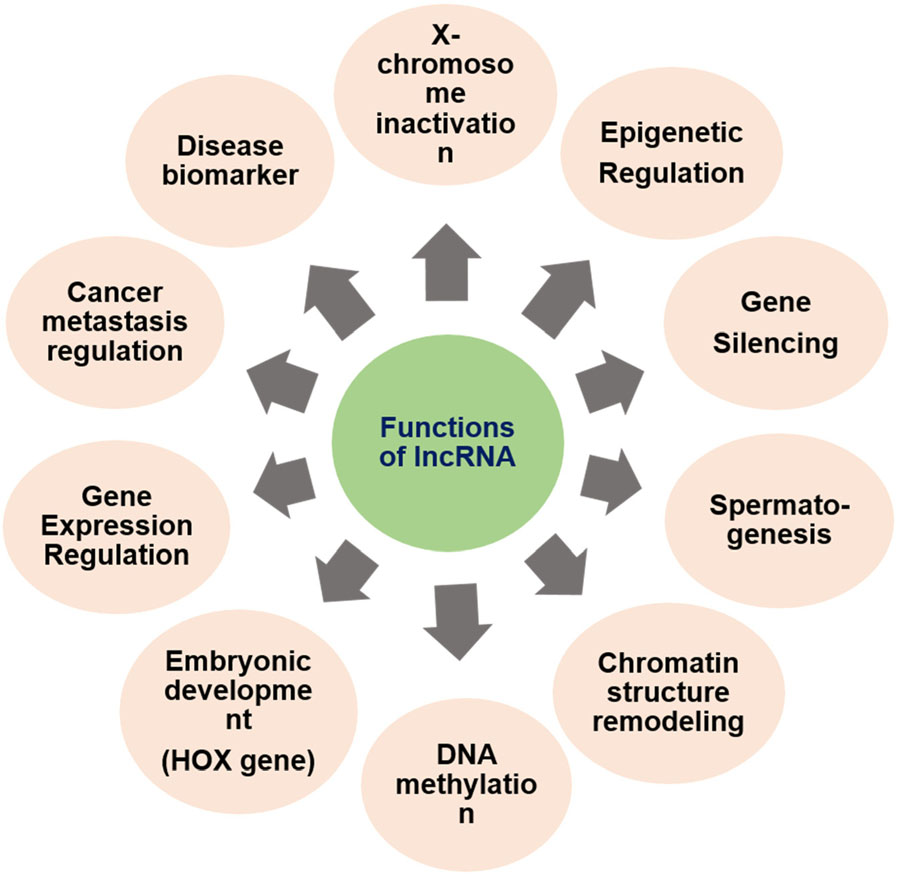
Various Functions of lncRNAs in biological processes

The lncRNAs play a versatile role, as discussed above, in various biological processes and disease states by interacting with DNA, RNA and other transcriptional molecules. They have a role in histone modification, chromatin remodeling, gene expression regulation, gene silencing, DNA methylation, heat shock response and embryogenesis [3]. The mutation in lncRNA is associated with various diseases including virus infection, cancer, and neurodegenerative disorders. Any dysregulation in lncRNAs influences the normal cellular functions including cell proliferation, resistance to apoptosis, induction of angiogenesis and evasion of tumor suppressors [28] (Fig. 2). Knockdown of some lncRNAsis responsible for the change in gene expression due to loss of pluripotency[86] of embryonic cells. Till date, very sparse research has been conducted on lncRNAs. Divergent lncRNAs has been adumbrated in Table 1 with their roles in normal physiological and pathological conditions in different mammals.

**Table 1:** List of some Long non-coding RNAs and their role in different tissues

SN	Type or Family	Target Tissue	Role	Reference
1	Heat shock RNA1 (HSR1)	Various tissues	• stimulates trimerization of heat-shock factor 1 (HSF1)with eukaryotic translation elongation factor 1A	[91]
2	Meiotic recombination hot spot1 locus (Mrhl)	Located in nucleus	• Regulate spermatogenesis	[23]
3	HongrES2	Expressed specifically in the cauda-epididymis	• The transcript *mil-HongrES2* inhibits expression of an epididymis-specific protein CES7 and its cholesterol esterase activity; • its overexpression results in retarded sperm capacitation	[70]
4	Testis-specific X-linked (Tsx)	Expressed in pachytene spermatocytes	• regulatory role in germline meiotic division	[1]
5	Dmrt1-related gene (Dmr)	Testis-specific	• essential transcription factor that promotesspermatogonial development by up-regulating Sohlh1 (Spermatogenesis and Oogenesis Specific Basic Helix-Loop-Helix 1); • prevents premature meiosis in spermatogonia by repressing Stra8 (Gene stimulated by retinoic acid 8)	[76]
6	Homeobox antisense intergenic RNA (HOTAIR)	Gastric adenocarcinoma tissues, Lung, Breast, Kidney	• Promotes cancer cell migration, invasion, and metastasis • Increased expression may affect genomic relocalization of the polycomb repressive complex 2; • Can enhance trimethylation of H3K27; • biomarker for poor prognosis in colorectal cancer	[32, 48]
7	Metastasis-associated lung adenocarcinoma transcript 1 (MALAT1)	Lung, pancreas	• Promotes cancer cell migration, invasion, and metastasis • Knockdown of MALAT1 (in HCC cell line) demonstrated amarked reduction in tumor progression. So it can be used as negative prognostic biomarker	[29]
8	Maternally Expressed Gene (MEG3)	Glioma cells, bladder, gastric tissues	• Tumor suppressive • Due to down-regulated expression in various tumor conditions including meningioma and glioma, it acts as tumor suppressor	[104]
9	Taurineupregulated gene 1 (TUG1)	Osteosarcoma tissue	• Tumor suppressive • Inhibit apoptosis	[115]
10	GAS5	Lung, breast, colorectal, kidney, prostate	• Induces apoptosis and suppresses miR-21 expression	[92]
11	BRAF Activated Noncoding RNA (BANCR)	Lung	• Tumor suppressive	[96]
12	H19	Lung	• Tumor suppressor gene • Higher H19 expression due to demethylation of promoter region results in induction of lung cancer	[12, 77]

### The role of lncRNA in epigenetics

In the early 1990s it was discovered that lncRNAs are involved in epigenetic gene regulation (viz. *H19* and *Xist*) [111]. In this section the lncRNA genes viz. Xist and H19 that are associated with epigenetic regulation of pathophysiological conditions are discussed.

#### Xist

One of the X-chromosomes in female mammals gets randomly inactivated (heterochromatinizedfacultative) during early embryonic stages to ensure dosage compensation in females with regard to the hemizygotic males harboring a single copy of those X-linked genes. X-inactive specific transcript (*Xist*), a 17 Kb gene located on mammalian X-chromosome, is an example of lncRNA which is responsible for X-chromosome inactivation in eutherian mammals. The regulation of cis-X inactivation is initiated by coating the X-chromosome and engaging polycomb repressive 2 (PRC2) complex to specific sites. This results in histone H3 lysine K27 trimethylation (H3K27me3) and X-linked inactivation [111].

#### H19

This lncRNA gene is located at Beckwith-Wiedemann Syndrome (BWS) locus in humans [68]. The lncRNA H19, not only regulates maternal imprinting during embryogenesis but also binds to methyl-CpG-binding-domain protein 1 (MBD1) and recruits histone-lysine-methyltransferase-containing complexes to place repressive H3K9 methylation marks on target imprinted loci [88]. HuR, an RNA binding protein, negatively regulates the expression of miR-675 by binding with *H19* and is responsible for decreased cell proliferation and limited placental growth before birth [45].

### Genomic imprinting

Genomic imprinting is an epigenetic process by which a specific gene is expressed in a monoallelic manner depending on the parent of origin [100]. The lncRNAs were also found to be involved in some imprinting processes. In the process of uniparental gene expression, the lncRNA recruits DNA methyltransferases instead of PRC2 for histone modification and DNA methylation [67]. The orthologs of some human lncRNAs (Airn, H19, Kcnq1ot1, Meg3, and Meg8) have been identified in 24 different species that are responsible for controlling genomic imprinting [44]. The lncRNAsAirn and Kcnq1 opposite transcript 1 (Kcnq1ot1)/ long QT intronic transcript 1(L1 T1) are responsible for suppression of paternally inherited genes [103]. The clusters of imprinted genes are found to be conserved containing at least one lncRNA gene. These lncRNAs form a cluster with DNA duplex to produce a triplex structure [99]. Insulin-like growth factor-2 (Igf2) and insulin-like growth factor-2 receptor (Igf2r) are examples of maternally and paternally imprinted genes, respectively, for embryonic growth control [44]. Mental disorder or incidence of cancer has been associated with dysregulated imprinting of such genes [42].

### Cancer

The study of lncRNAs till date inferences that whether these molecules are associated and involved in various biological processes but their dysregulation can develop cancer. Metastasis-associated lung adenocarcinoma transcript 1 (Malat1) is a lncRNA that is involved in localization of splicing factors serine/arginine to the nuclear speckles. These lncRNAs control the alternative splicing of various mRNA precursors and play important role in the pathogenesis resulting from metastasis and cell invasion [41]. It can affect regulation of cytoskeletal and extracellular matrix genes at transcriptional and post-transcriptional levels [98]. Some transcripts of lncRNA including *Xist* and *Kcnq1ot1* are also involved in dosage compensation [24]. In another report, it has been mentioned that the apoptosis of breast cancer cells can be inhibited by plasmacytoma variant translocation 1 gene (PVT1) [101, 102, 105].

HOTAIR lncRNA can promote cancer metastasis in the chromatin state of cancer cells through epigenetic variations [39]. The up-regulation of HOTAIR could be associated with poor or failed prognosis, in various types of cancers including breast, liver, gastrointestinal and pancreatic cancers [27, 107]. Steroid receptor RNA activator (SRA) is a lncRNA that is linked to breast cancer with highly conserved helices and loops [72]. PCAT-1 is another lncRNA involved in the stimulation of cell proliferation [83]. Prostate cancer has been associated with over-expression of long intergenic non-protein coding RNA gene SCHLAP1 (SWI/SNF Complex Antagonist Associated with Prostate Cancer 1) [89]. Up-regulation of TUG1 promotes proliferation and migration of esophageal squamous cell carcinoma while its down-regulation inhibits osteosarcoma cell proliferation and promotes apoptosis [94].

### Autoimmune disease

An abnormal immune response to the normal body due to complex environmental profile results in the development of autoimmune disease(s). Research reports show that lncRNAs contribute to the development of certain autoimmune diseases in human and mice, in a similar manner of some of the miRNAs that are found to be essential for normal immune response and to prevent autoimmune diseases [93]. Autoimmune diseases caused by a minute change in gene regulation or cells related to immune response (B cells, T cells etc) [110] can be detected from the regulation process of lncRNAs. The up or down-regulation of some lncRNAs has also been detected to be associated with various autoimmune diseases. For example, the up-regulation of *GAS5* is associated with sclerosis and tuberculosis whereas down-regulation was detected in rheumatoid arthritis [65]. Under homeostatic conditions, lnc13 is known to repress the expression of inflammation-related genes [7].

### Differentiation and regulation of spermatogonial stem cells (SSC)

Another role of lncRNA has been detected in sexual identities via regulating the expression of sex determination genes from fly to mice [90]. The role of lncRNAs in differentiation and regulation of SSC self-renewal has been reported in glial cell-derived neurotrophic factor (GDNF) [58, 59]. Some lncRNAs are also involved in regulation of male reproduction. In vitro study in mouse showed that AK015322 lncRNA promotes proliferation of spermatogonial stem cell line C18–4 [37].

### Role in spermatogenesis

The whole genome expression profile of spermatogenesis-related lncRNAs has revealed that testis is one of the highly abundant lncRNA containing tissue. The expression of testis-specific lncRNAs, *lncRNA-Tcam1* and *lncRNA-HSVIII* has been identified in spermatocyte stages [114]. TUG1 is a long, intergenic gene that is up-regulated in various human cell lines and tumors.

## Other physiological functions

Meiotic recombination hot spot locus (Mrhl) is a 2.4-kb mono-exonic lncRNA sited in the nucleus of mouse chromosome 8 which play a major role in the regulation of spermatogenesis. Despite that HongrES2 (in rat), Testis-specific X-linked (Tsx) (expressed in pachytene spermatocytes), the Dmrt1-related gene (Dmr) (testis-specific lncRNA) are some other lncRNA identified in different species and tissues [62]. During the embryonic development of mouse, some lncRNA including AB063319, AK003491, and AK044800 have been reported to be abundantly expressed in brain, muscle, liver, lung and neuroendocrine tissues [8].

## The lncRNAs as biomarkers for disease control

The lncRNAs are involved in various biological and pathological processes including neurogenesis, oncogenesis and stem cell pluripotency[103]. Various lncRNAsare known to possess tumor suppressive and oncogenic roles thus can act as a biomarker for disease diagnosis. At present, there are limited reports on lncRNAs as validated biomarkers. The altered lncRNA expression pattern in uterine corpus endometrial carcinoma (UCEC) suggested that lncRNAs can act as predictive biomarkers for a high-risk patient with endometrial carcinoma [101, 102, 105]. Highly up-regulated in liver cancer (HULC) has been found to act as a diagnostic marker for hepatocellular cancer [2]. The level of PCAT1 in urine can help in detection of poor prognosis prostate cancer patients [41]. HOTAIR, MALAT1, microvascular invasion of HCC (MVIH), H19 etc. are some other examples of lncRNA that can be used as biomarkers. Alternatively, DD3 can be used as a negative prognostic biomarker in prostate cancer [13]. The lncRNAs can also be used as biomarkers for sepsis detection in patients [22]. They are also involved in the pathogenesis of ovarian cancer [84].

## Databases for lncRNAs

The lncRNA-pool in the genome can be bio-computationally predicted, identified and finally validated through various experimental and computational methods including microarray, SAGE, RNA-immuno-precipitation RNA-Seq, in silico identification of open reading frame (ORF) and by machine learning techniques [63]. The data obtained from the de novo analysis is further organized through the specific database. The database can maintain, archive or retrieve the information related to lncRNAs. This can further annotate the features and will provide the interactions and functions with different molecules in systems biology. To study the structure and function of different lncRNAs, various online databases have been identified to date. Some of them are mentioned here with some descriptions. lncRNAdb (http://www.lncRNAdb.org/) provides detailed information about functional lncRNAs[9]. The sequence and structure information of human lncRNA is available through LNCipedia (http://www.lncipedia.org) [112]. ChIPBase (http://rna.sysu.edu.cn/chipbase/) database helps to study transcription factor binding site and motifs and provides the decoded information of transcriptional regulatory network [18]. The study of the expression of human and mouse lncRNA is available through a chip-based strategy of NRED (http://nred.matticklab.com/cgi-bin/ncrnadb.pl) [97].

## Conclusion

Significant research endeavors are being exercised on non-coding RNAs (ncRNAs)with an aim to study their role in biological processes, apply ncRNAs as biomarkers and to unveil the systems biology. A decade ago, miRNAs were the most popular ncRNA for scientific research and study but now the other class of ncRNAs, lncRNAs is also on the high priority to unveil their role in disease development and control process. The available literature shows their important contribution in metastasis and thus can be a target in cancer therapy. They can be used as disease biomarker and to explore systems biology. Last but not the least, the transcription machinery of eukaryotic cells is partially depicted by the major players like coding and non-coding RNAs. Recent studies at Buratowski laboratory of Harvand Medical School (https://buratowski.hms.harvard.edu/) has postulated and experimentally validated that the transcription process itself can modify the chromatin that underscores the importance of several factors other than noncoding RNAs like lncRNA [46]. In depth studies are warranted to unveil the complete systems biology involved in modulation of gene expression in eukaryotic cells.
